# Neuromuscular fatigue in men and women during severe-intensity exercise

**DOI:** 10.1590/1414-431X2025e14448

**Published:** 2025-05-09

**Authors:** G. Cristina-Souza, J.C. Schamne, P. Souza-Santos, A.C. Santos-Mariano, D.B. Coelho, R. Bertuzzi, A.E. Lima-Silva, A.H. Marinho

**Affiliations:** 1 Grupo de Pesquisa em Exercício e Nutrição, Universidade do Estado de Minas Gerais, Poços de Caldas, MG, Brasil; 2 Grupo de Pesquisa em Performance Humana, Universidade Tecnológica Federal do Paraná, Curitiba, PR, Brasil; 3 Engenharia Biomédica, Universidade Federal do ABC, São Paulo, SP, Brasil; 4 Grupo de Pesquisa em Desempenho Aeróbio, Universidade de São Paulo, São Paulo, SP, Brasil; 5 Laboratório de Ciências Aplicadas ao Esporte, Instituto de Educação Física e Esporte, Universidade Federal de Alagoas, Maceió, AL, Brasil

**Keywords:** Endurance performance, Exhaustion, Central fatigue, Muscle activation, Peripheral fatigue

## Abstract

The aim of this study was to explore sex differences in neuromuscular fatigue during a severe-intensity cycling exercise. Twenty-four healthy participants (12 women and 12 men) cycled at 80% of the difference between gas exchange threshold and maximal power output to the limit of tolerance. Neuromuscular fatigue was assessed by the decrease in maximal voluntary contraction of the knee extensors before and after exercise, and central and peripheral fatigue was measured by the decrease in voluntary activation and quadriceps potentiated twitch force before and after exercise. Women presented shorter time to task failure (P=0.025) and lower levels of neuromuscular fatigue (P=0.006) and peripheral fatigue (P<0.001) than men. Women and men showed different patterns of muscle activation during exercise, with women presenting greater muscle activation at the beginning of exercise and sustaining this elevated muscle activation throughout exercise, while men increased muscle activation from the beginning to the end of exercise. In conclusion, women had lower levels of neuromuscular fatigue, mainly caused by lower levels of peripheral fatigue, and a different muscle activation pattern in an exhaustive severe-intensity cycling exercise.

## Introduction

Recent data indicate that only 34% of the studies conducted in sport and exercise science between 2014 and 2020 included women in the sample, with only 6% of these studies having exclusively women as participants (1). This reveals that women are under-represented in sport and exercise science research and that studies exploring sex differences are essential for a better understanding of the similarities and differences between men and women during exercise.

Men and women exhibit physiological and anatomical differences that influence their physiological adjustments during exercise (2). For example, the exercise-induced vasodilatory response of the femoral artery (3) and capillary density per unit of skeletal muscle in the *vastus lateralis* (4) are higher in women than in men. Women also present a greater proportion of more fatigue-resistant type I muscle fibers in the *vastus lateralis* (4) and higher muscle oxygenation during exercise (5,6) than men. These particularities of female physiology might result in a different degree of neuromuscular fatigue after exercise. A few studies were recently conducted to compare sex differences in the level of end-exercise neuromuscular fatigue after a cycling exercise performed in the severe-intensity domain (i.e., above critical power) (6-9). These studies, however, reported conflicting results, probably due to different methodological approaches. For example, one study demonstrated no differences between men and women for neuromuscular fatigue after cycling to task failure at 80% of peak power output (7), with the task-to-failure trial being preceded by a 30-min constant-load cycling exercise at a power output around maximal lactate steady-state. A follow-up study of the same group, however, demonstrated that women presented lower levels of end-exercise neuromuscular fatigue when cycling at a power output slightly above the maximal lactate steady-state to task failure (8). The main cause of the lower level of neuromuscular fatigue in women was a lower impairment in contractile function when compared to men (8). Similarly, a study reported lower levels of peripheral but not central fatigue in women than men after a severe-intensity cycling exercise (110% of critical power) performed to task failure (6). Another study, however, demonstrated lower levels of neuromuscular fatigue in women than in men at task failure of a severe-intensity cycling exercise (90% of V̇O_2max_; maximum oxygen uptake), but the peripheral factors underpinning this lower neuromuscular fatigue were not evident (9). In addition, as central fatigue was not measured, it was not possible to ascertain whether the lower levels of neuromuscular fatigue observed in women was a consequence of lower central fatigue. The different forms to determine exercise intensity (6-9), changes in exercise intensity throughout the trial (7), and the absence of simultaneous assessment of central and peripheral fatigue (9) in the aforementioned studies might explain these conflicting results and preclude a deeper understanding of possible sex differences in the degree of neuromuscular fatigue and its central and peripheral determinants, particularly during severe-intensity exercise.

In this study, we explored sex differences in neuromuscular fatigue during a severe-intensity cycling exercise performed to the limit of tolerance. By fixing exercise intensity within the severe-intensity domain (i.e., constant-load trial) and integrating central and peripheral fatigue measurements, we provide new insights into sex differences in neuromuscular fatigue. We hypothesized that, compared to men, women would have lower fatigability due to lower levels of peripheral fatigue.

## Material and Methods

### Participants

Twelve healthy men and twelve healthy women, who were engaged in regular general training programs for developing physical fitness (running, cycling, and resistance training), volunteered to participate in this study (Table 1). The required sample size was calculated inputting the following parameters on GPower software (version 3.1.9.7, Germany): 1) effect size of 0.38 based on the expected difference in peripheral fatigue between women and men after a severe-intensity exercise (5); 2) alpha of 0.05; 3) power (1-ß) of 0.95; 4) two groups (i.e., men and women); 5) two measurements (pre- and post-exercise); 6) correlation among repeated measures of 0.5; and 7) nonsphericity correlation of 1. This calculation returned a required sample size of 24 participants (12 men and 12 women).

**Table 1:** Demographic characteristics of participants.

	Men (n=12)	Women (n=12)	P-value	Cohen's d	95%CI
Age (years)	25.8±6.0	25.2±3.6	0.350	0.12	[-3.87, 4.53]
Body mass (kg)	80.1±11.7	60.5±5.7	**<0.001**	**2.13**	**[-27.47, -11.83]**
Height (cm)	174.4±10.0	162.1±5.5	**0.001**	**1.52**	**[-19.14, -5.44]**
Body fat (%)	10.0±4.1	22.8±3.4	**<0.001**	**3.39**	**[9.59, 16.02]**
PO_peak_ (W)	290.3±26.9	195.3±27.0	**<0.001**	**3.52**	**[-117.89, -72,15]**
PO_peak_ (W/kg)	3.7±0.5	3.2±0.4	**0.041**	**0.97**	**[-0.87, -0.02]**
GET (W)	120.8±23.4	116.7±19.5	0.707	0.19	[-22.40, 14.07]
GET (W/kg)	1.5±0.3	1.9±0.3	**0.004**	**1.33**	**[0.15, 0.68]**
V̇O_2peak_ (L/min)	3.4±0.3	2.2±0.4	**<0.001**	**3.39**	**[-1.49, -0.90]**
V̇O_2peak_ (mL·kg^-1^·min^-1^)	43.4±7.2	37.4±4.9	**0.027**	**0.97**	**[-11.16, -0.74]**
RER_peak_	1.2±0.1	1.3±0.1	0.686	1.00	[-0.05, 0.07]
HR_peak_ (beats/min)	180±13	180±7	0.919	0.00	[-10.68, 9.68]
RPE_peak_	19.8±0.4	19.2±1.0	0.109	0.79	[-1.30, 0.06]

PO_peak_: peak power output; GET: gas exchange threshold; V̇O_2peak_: peak oxygen uptake; RER_peak_: peak respiratory exchange ratio; HR_peak_: peak heart rate; RPE_peak_: peak rating of perceived exertion; CI: confidence interval. Data are reported as means and SD. Values in bold indicate significant differences between men and women (P<0.05; Student's *t*-test).

The inclusion criteria were: 1) age between 18 and 40 years; 2) free of neuromuscular and cardiovascular dysfunctions; 3) no use of exogenous anabolic androgenic steroids, medicines, or nutritional supplements that could influence physical performance; 4) training frequency of at least 3 times per week (minimum of 60 min per session); and 5) previous experience with bicycle or cycle ergometer exercise for at least the previous six months. The last two inclusion criteria were adopted to recruit women and men with similar level of sex-specific physical fitness and thus avoid potential confounding influence of exercise capacity on neuromuscular fatigue (10). Additionally, women were required to have a regular menstrual cycle, without any signs of menstrual disorders in the six months before the study. Participants were informed about the procedures, risks, and benefits associated with the experimental study, and signed a written informed consent form. The study was conducted according to the Declaration of Helsinki and approved by the Research Ethics Committee of the Federal University of Paraná.

### Experimental design

Participants visited the laboratory on three different days, with a minimum of 72 h and a maximum of one week between visits. On the first visit, body mass, height, and skinfolds (chest, abdomen, and thigh for men and triceps, suprailiac, and thigh for women) were measured to determine the body fat percentage using standardized equations (11). Subsequently, participants performed a graded exercise test on an electromagnetically-braked cycle ergometer (Ergo-Fit, Germany) to determine their gas exchange threshold (GET), peak power output (PO_peak_), and peak of oxygen uptake (V̇O_2peak_). On the second visit, participants were familiarized with all experimental procedures, which included a severe-intensity cycling exercise performed to task failure and pre- and post-exercise neuromuscular function assessment. On the third visit, in a 2-h postprandial state, participants performed the experimental trial, which consisted of a severe-intensity cycling exercise performed to task failure, with pre- and post-exercise neuromuscular function assessment.

Participants were instructed to refrain from exhaustive exercise, alcohol, and food or supplements containing caffeine during the 24 h before the experimental trials. The calendar counting method was used to determine the phases of the menstrual cycle, with women participants performing the experimental trial between late-follicular and mid-luteal phases (12). The experimental trial visit was rescheduled if the planned day coincided with the early-follicular (i.e., during menses) or late-luteal (i.e., before the onset of menses) phases, or when there were residual symptoms associated with the onset of menses (13).

### Maximal graded exercise test

Participants performed a maximal graded exercise test (5-min warm up at 50 W followed by increments of 25 W/min) maintaining a pedal frequency between 70 and 80 revolutions per minute throughout the test. Task failure was assumed when participants were unable to maintain cadence above 70 revolutions per minute even with verbal encouragement, or by voluntary disengagement. Pulmonary and metabolic parameters, heart rate (HR), and rating of perceived exertion (RPE) were monitored throughout the test.

For further determination of the exercise intensity corresponding to the severe-intensity domain, GET was visually identified by two independent investigators from the first disproportionate increase in carbon dioxide production, an increase in ventilatory equivalent for oxygen uptake without an increase in ventilatory equivalent for carbon dioxide production, and an increase in end-tidal oxygen pressure with no fall in end-tidal carbon dioxide pressure (14). In addition, PO_peak_ was determined as the power during the final complete stage; when the final stage was incomplete, PO_peak_ was determined by multiplying the fractional time sustained in the final incomplete stage and the increment rate. The work rate of the experimental trial was set at 80% of the difference between the GET and PO_peak_ (Δ80), an exercise intensity that is in the severe-intensity domain (15).

### Experimental trial

As a warm-up, participants cycled at 90% of their GET for five minutes and performed four 5-s isometric contractions of knee extensors at 60, 70, 80, and 100% of the maximal isometric voluntary contraction (MVC), interspersed by a 30-s rest between contractions. Participants then performed three MVCs (60-s rest interval between contractions) with electrical stimulation of the femoral nerve during and immediately after each MVC for neuromuscular function assessment (pre-exercise assessment). Participants then initiated a cycling exercise to task failure at Δ80, maintaining pedal cadence between 70 and 80 revolutions per minute throughout the trial, with criteria for task failure being the same as described for the maximal graded exercise test. Pulmonary and metabolic parameters, HR, and RPE were monitored during the cycling exercise. The surface electromyography signal of the right *vastus lateralis* was also recorded at the beginning and end of the cycling exercise. The neuromuscular function was reassessed one minute after the cycling exercise (post-exercise measurement).

### Data collection

#### Neuromuscular function assessment

Details of the procedures for neuromuscular function assessment have been reported previously (15). Briefly, the skin where the electrodes were attached for nerve stimulation and recording was shaved and cleaned with alcohol to reduce impedance to <10 kΩ. Then, a monopolar cathode electrode (Ambu^®^ Neuroline 715, Denmark) was fixed on the right inguinal triangle for electrical stimulation of the femoral nerve, and an anode electrode was fixed on the gluteal fold. A bipolar electrode with full-surface solid adhesive hydrogel (Ambu Neuroline 715) was fixed over the *vastus lateralis* of the right thigh and a ground electrode was fixed on the tibia according to the Surface Electromyography for the Non-Invasive Assessment of Muscles Standards (16) to ensure the optimized recording of electromyography and peak-to-peak amplitude of the compound muscle action potential of the *vastus lateralis* (M_wave_). The position of each electrode was also marked with permanent ink to ensure identical placement during all trials.

Participants sat on a custom-made knee extensor chair (hip at 120° and knees at 90°), with a calibrated load cell attached to their right ankle via a cuff (EMG System, Brazil). The cathode electrode was moved around the femoral triangle and percutaneous electrical nerve stimulation was performed using an electrical stimulator (Neuro-TES, Neurosoft, Russia) to determine the position that elicited the greatest values of quadriceps twitch force and M_wave_. The position of the electrodes was marked with permanent ink to ensure identical placement in all experimental trials. Then, the optimal stimulation intensity for neuromuscular function assessment was determined by percutaneous electrical nerve stimuli on the femoral nerve. The electrical stimulator delivered a square-wave, 1-ms duration single-pulse (1 Hz) stimulus on the femoral nerve, starting at 15 mA and increasing by 5 mA (30-s rest between stimuli) to a plateau in the quadriceps twitch force and M_wave_. To ensure maximal stimulation when assessing pre- and post-exercise neuromuscular function, the stimulation intensity was set at 120% of the plateau of the quadriceps twitch force and M_wave_.

To assess pre-exercise neuromuscular function, the plateau of the quadriceps twitch force and M_wave_ were checked, and participants performed three 5-s MVCs (60-s rest between contractions). A single electrical stimulus was applied as soon as the plateau of maximal force was reached (17), which occurred in approximately 2-3 s after the beginning of voluntary contraction. Potentiated quadriceps twitch force evoked by single (Q_twpot_) and paired pulses at 10 Hz (Q_tw10_) and 100 Hz (Q_tw100_) was obtained 2, 4, and 6 s after the MVC, respectively. This procedure was repeated post-exercise, but only one MVC and associated electrical stimuli was performed to avoid influence of recovery on neuromuscular function (18).

#### Cardiopulmonary and metabolic measurements

Pulmonary and metabolic parameters were measured breath-by-breath during the maximal graded exercise test and experimental trial using an automatic metabolic cart (Vmax Encore, Carefusion, USA). The metabolic cart was calibrated before each trial using a 3-L syringe, ambient air, and two cylinders containing air of known oxygen and carbon dioxide concentrations (16% oxygen and 4% carbon dioxide in one cylinder and 26% oxygen and 0% carbon dioxide in another). HR was also recorded continuously using an HR monitor (Polar FT1 Coded, Finland). RPE was recorded using a 15-point Borg scale (19).

#### Electromyography recording

The surface electromyography signal of the *vastus lateralis* was recorded during MVCs and throughout the exercise trial at a sampling rate of 20,000 Hz (Neuro-MEP-Micro, Neurosoft) and amplified with an octal bio-amplifier (input impedance=1 GΩ, common mode rejection ratio=100 dB, gain=2000).

### Data analyses

#### Neuromuscular fatigue

The MVC was determined as the 250-ms average force around the peak force reached before the superimposed twitch (i.e., during the first 2-3 s of the voluntary contraction) (20). The Q_twpot_, Q_tw10_, and Q_tw100_ were the peak forces attained after the corresponding electrical stimulation. M_wave_ peak-to-peak amplitude was calculated from the *vastus lateralis* surface electromyography signal after the 1 Hz stimulation (18). Voluntary activation (VA) was calculated using a modified version of the superimposed twitch formula (Equation 1) (21):

$$VA\%=100-D\times \left(\frac{F_{b}}{MVC}\right)/Ptw-pot\times 100$$ [Eq. 1]

where F_b_ is the voluntary force immediately before the superimposed twitch, D is the difference between F_b_ and maximum force evoked by the superimposed twitch, and MVC is the maximal voluntary contraction.

The average of the three pre-exercise measures of MVC, Q_twpot_, Q_tw10_, Q_tw100_, VA, and M_wave_ were used as pre-exercise measurements (15). The pre-to-post exercise change in MVC was used as an index of neuromuscular fatigue (18), while changes in the Q_twpot_ and VA were used as markers of peripheral and central fatigue, respectively (18). The pre-to-post exercise changes in the Q_tw10_ and the Q_tw10_:Q_tw100_ ratio were used as indices of low-frequency fatigue and changes in the Q_tw100_ as markers of high-frequency fatigue (18). The pre-to-post exercise change in M_wave_ amplitude was used as a marker of alterations in membrane excitability (18).

#### Cardiopulmonary and metabolic responses

The average V̇O_2_ and respiratory exchange ratio (RER) of the last 10 s of the maximal graded exercise test was assumed as V̇O_2peak_ and RER_peak_, respectively. The average HR of the last 5 s of the maximal graded exercise test was assumed as HR_peak_. The highest RPE of the maximal graded exercise test was assumed as RPE_peak_. The attainment of maximal effort was confirmed when two or more of the following criteria were met: an increase in V̇O_2_ of less than 2.1 mL·kg^-1^·min^-1^ between two consecutive stages, a RER_peak_ greater than 1.1, and the attainment of a HR_peak_ greater than 90% of the predicted maximal HR (i.e., 220-age) (22). Corresponding measures during the severe-intensity cycling exercise was named as V̇O_2end_, RER_end_, HR_end_, and RPE_end_, respectively. These parameters were compared between men and women to determine potential sex differences in the effort employed during the exercise tasks.

#### Electromyography

The raw surface electromyography signal during the severe-intensity cycling exercise was filtered with a second-order Butterworth band-pass filter (cutoff frequencies set at 20 and 500 Hz). The root mean square (RMS) of each burst was calculated as previously described (15). The RMS from 10 to 20 s at the beginning and during the last 10 s of the trial (excluding the last three contractions to avoid any interference of anticipated reduction in cadence at task failure) were averaged and normalized to the RMS mean in the three pre-exercise MVCs (%RMS_MVC_) to explore potential sex differences in muscle activation during the cycling exercise (15).

### Statistical analyses

Data are reported as means±SD unless otherwise indicated. The Shapiro-Wilk test was used to check data distribution. All variables were normally distributed, except for height, GET, RPE_peak_, and RPE_end_. Thus, normally distributed variables were compared between men and women using an independent *t*-test and non-normally distributed variables using Mann-Whitney U test. The Cohen's d effect size was also calculated using means and pooled standard deviation, where values <0.20 indicates small effect, between 0.20 and 0.39 indicates moderate effect, and ≥0.40 indicates large effect (23). A two-way mixed analysis of variance was used to determine the effects of group and exercise time on %RMS_MVC_ and parameters of neuromuscular function. The sphericity of the variances was checked via the Mauchly test, with the Greenhouse-Geisser correction applied when sphericity was violated. If significant main effects were found, the Duncan *post hoc* test was applied to locate the differences. Partial eta squared (η_p_^2^) for main and interaction effects was also calculated. The η_p_^2^ was considered as small (η_p_^2^<0.06), moderate (0.06≤η_p_^2^<0.15), or large (η_p_^2^≥0.15) (23). Significance was assumed when P<0.05. All analyses were performed using the statistical software Statistica (version 10, StataSoft Inc.^®^, USA).

## Results

As expected, women were shorter, lighter, and had a higher body fat percentage than men. Women also had lower absolute and relative PO_peak_ and V̇O_2peak_ than men. The relative, but not absolute GET, was higher in women than in men. There were no differences between men and women for RER_peak_, HR_peak_, and RPE_peak_ (Table 1).

Time to task failure, power at Δ80, and absolute and relative V̇O_2end_ were lower in women than in men, while RER_end_, HR_end_, and RPE_end_ were similar for men and women. Likewise, V̇O_2end_, HR_end_, and RPE_end_, when reported as a percentage of their peak values obtained in the maximal graded exercise test, were similar for men and women (Table 2).

**Table 2:** Endurance performance and cardiopulmonary and metabolic parameters during a severe-intensity cycling exercise performed at a power output of 80% of the difference between gas exchange threshold and maximal power output to task failure in men and women.

	Men (n=12)	Women (n=12)	P-value	Cohen's d	95%CI
Time to task failure (s)	328.6±56.0	262.3±76.0	**0.025**	**0.99**	**[-123.31, -9.34]**
PO_Δ80_ (W)	256.4±24.0	179.5±23.6	**<0.001**	**3.23**	**[-97.05, -56.75]**
V̇O_2end_ (L/min)	3.4±0.2	2.3±0.4	**<0.001**	**3.48**	**[-1.39, -0.87]**
V̇O_2end_ (ml·kg^-1^·min^-1^)	43.6±6.7	37.6±4.4	**0.018**	**1.06**	**[-10.77, -1.14]**
V̇O_2end_ (%V̇O_2max_)	100.5±6.0	100.5±5.6	0.999	0.00	[-4.89, 4.89]
RER_end_	1.2±0.1	1.2±0.1	0.421	0.00	[-0.10, 0.04]
HR_end_ (beats/min)	178±10	176±6	0.569	0.24	[-9.99, 5.88]
HR_end_ (%HR_max_)	98.9±2.9	98.4±3.0	0.704	0.17	[-3.14, 2.16]
RPE_end_	19.0±1.0	19.3±0.8	0.159	0.33	[-0.21, 1.37]
RPE_end_ (%RPE_max_)	95.6±4.9	100.6±7.7	0.070	0.77	[-0.44, 10.48]

PO_Δ80_: power output of 80% of the difference between the gas exchange threshold and maximal power output_;_ V̇O_2end_: oxygen uptake at the end of exercise; V̇O_2max_: maximum oxygen uptake; RER_end_: respiratory exchange ratio at the end of exercise; HR_end_: heart rate at the end of exercise; RPE_end_: rating of perceived exertion at the end of exercise; CI: confidence interval. Data are reported as mean and SD. Values in bold indicate significant differences between men and women (P<0.05; Student's *t*-test).

**Figure 1:**
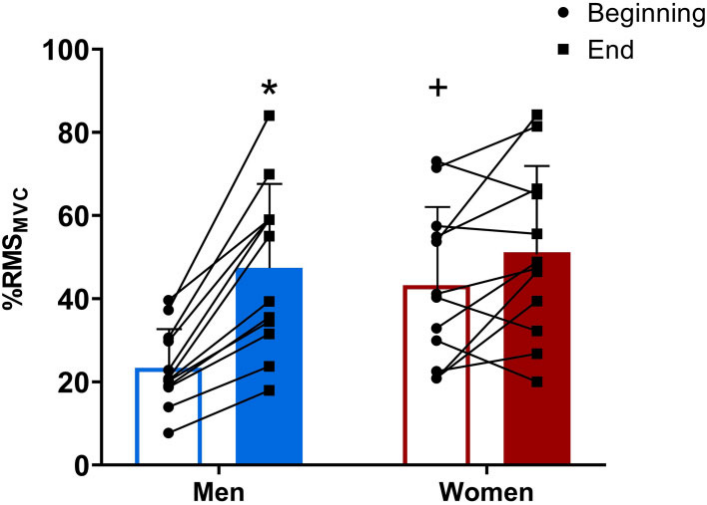
Electromyographic activity of the *vastus lateralis* (in percentage root mean square of pre-exercise maximal voluntary contraction, %RMS_MVC_) during a severe-intensity cycling exercise performed to task failure in men and women. Data are reported as mean and SD and individual data (dots). *P<0.01 *vs* beginning of exercise for the same group. ^+^P<0.01 compared to men at the same moment (two-way mixed ANOVA followed by Duncan *post hoc* test).

There was, however, a significant group×time interaction (P=0.006, η_p_^2^=0.296) for %RMS_MVC_ (Figure 1). Women presented a greater %RMS_MVC_ at the beginning of exercise than men (P=0.003). Women maintained this elevated muscle activation throughout the exercise by presenting no differences in %RMS_MVC_ between the beginning and end of the exercise (P=0.183). The %RMS_MVC_ increased from the beginning to end of the exercise in men (P<0.001).

**Figure 2:**
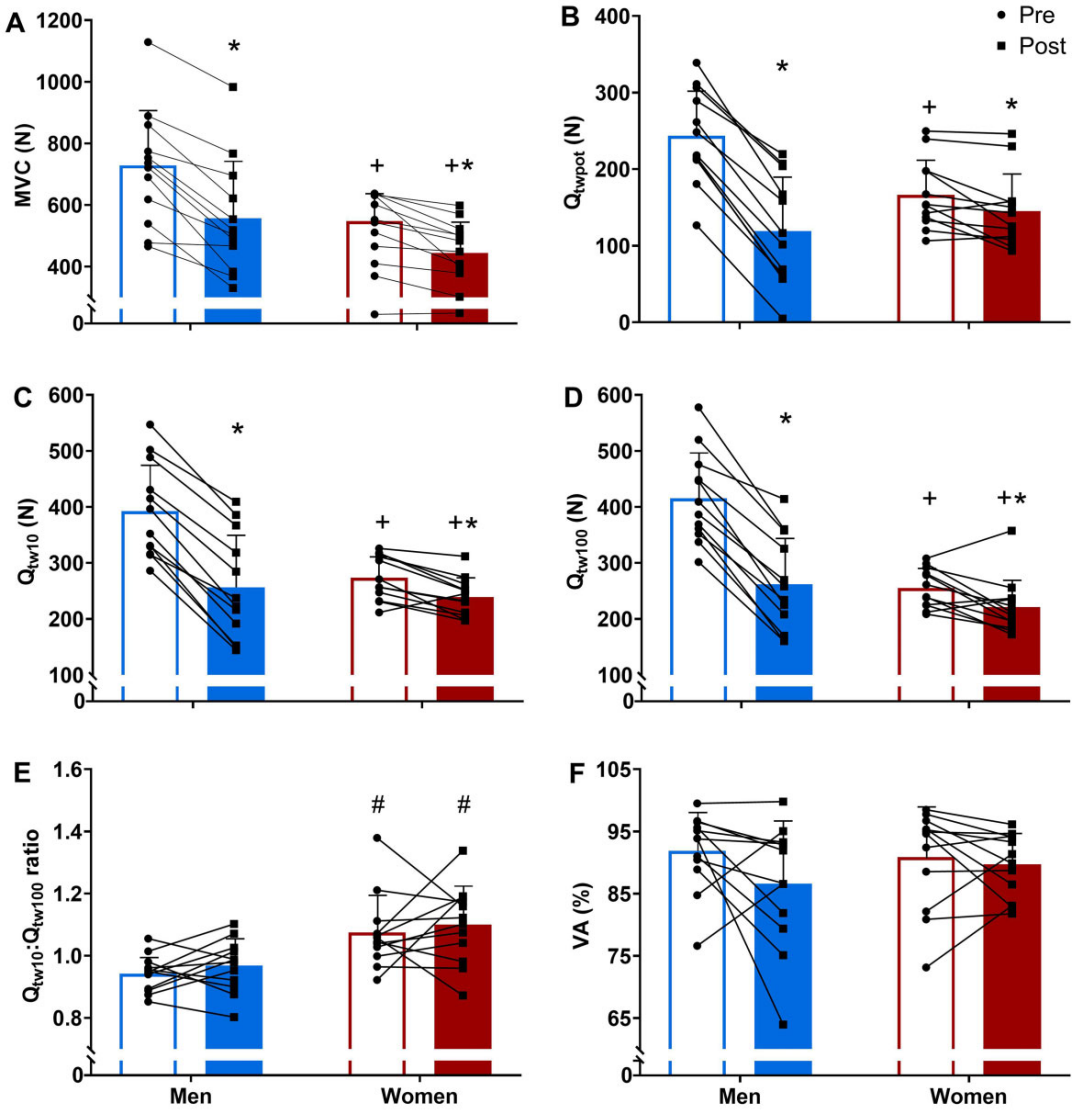
Maximum isometric voluntary contraction (MVC) (**A**), potentiated quadriceps twitch torque evoked by a single pulse (Q_twpot_) (**B**), paired pulses at 10 Hz (Q_tw10_) (**C**) and 100 Hz (Q_tw100_) (**D**), Q_tw10_:Q_tw100_ ratio (**E**), and voluntary activation (VA) (**F**) pre- and post-exercise of severe-intensity cycling performed to task failure in men and women. Data are reported as mean and SD and individual data (dots). *P<0.05 compared to pre-exercise in the same group. ^+^P<0.05 compared to men at the same moment. ^#^P<0.05 compared to men at the same moment (two-way mixed ANOVA followed by Duncan *post hoc* test).

**Figure 3:**
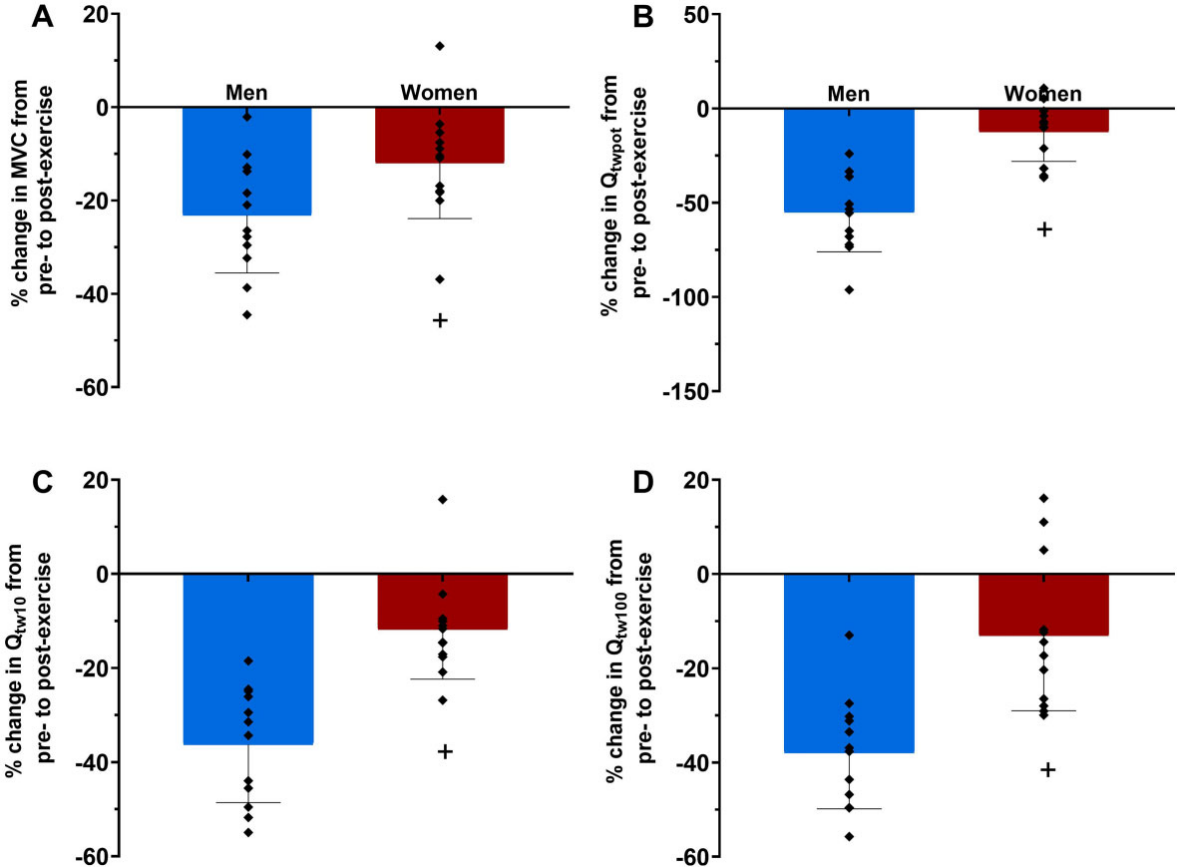
Pre- to post-exercise changes in maximum voluntary isometric contraction (MVC) (**A**), potentiated quadriceps twitch force evoked by single (Q_twpot_) (**B**), and paired pulses at 10 Hz (Q_tw10_) (**C**) and 100 Hz (Q_tw100_) (**D**) in men and women. Data are reported as means±SD and individual data. ^+^P<0.05 compared to men (Student’s *t*-test).

Women had lower MVC, Q_tw10_, and Q_tw100_, and a higher Q_tw10_:Q_tw100_ ratio than men (main effect of group, P<0.05, η_p_^2^=0.225, 0.219, 0.214, and 0.423, respectively), but there was no difference between women and men for Q_twpot_ (main effect of group, P=0.276; η_p_^2^=0.054). However, the magnitude of the pre-to-post exercise reduction in MVC, Q_twpot_, Q_tw10_, and Q_tw100_ was lower in women than in men (group×time interaction, P<0.05, η_p_^2^=0.299, 0.783, 0.749, 0.659, respectively; Figures 2A-D and 3A-D). There was no main effect of time or group×time interaction for the Q_tw10_:Q_tw100_ ratio (P>0.05, η_p_^2^=0.047 and 0.001, respectively; Figure 2E) and VA (P>0.05, η_p_^2^=0.121 and 0.054, respectively; Figure 2F). The M_wave_ was lower in women than in men (main effect of group, P=0.016, η_p_^2^=0.268), and increased from pre- (men: 20.8±4.3 mV and women: 16.7±2.5 mV) to post-exercise (men: 21.9±4.4 mV and women: 17.4±2.8 mV; main effect of time, P=0.008, η_p_^2^=0.316) regardless of group (group×time interaction, P=0.541, η_p_^2=^0.020).

## Discussion

In the present study, we explored potential sex differences in neuromuscular fatigue during a severe-intensity cycling exercise performed to the limit of tolerance. The main findings of the present study were: 1) women presented lower levels of neuromuscular fatigue, mainly caused by lower levels of peripheral fatigue, than men and 2) women and men presented different muscle activation patterns during exercise.

Compared to men, women experienced lower levels of neuromuscular fatigue after exercise, accompanied by lower levels of peripheral fatigue. Studies comparing neuromuscular fatigue in severe-intensity exercise performed to the limit of tolerance between women and men have reported conflicting results (6-9). While a previous study found no discernible differences between men and women in neuromuscular fatigue after cycling to task failure at 80% of PO_peak_ (preceded by 30 min cycling around maximal lactate steady state) (7), another study of the same group reported lower levels of post-exercise neuromuscular and peripheral fatigue after cycling slightly above the maximal lactate steady state to task failure in women than in men (8). In contrast, one study showed lower neuromuscular fatigue in women than in men after cycling at 90% of PO_peak_, but this lower level of neuromuscular fatigue was not accompanied by a lower level of peripheral fatigue (9), while another study found no differences in neuromuscular fatigue between women and men after cycling at 110% of critical power, but peripheral fatigue was lower in women than in men (6). It is difficult to explain these contradictory findings, but differences in statistical power or in exercise intensity set-up (percentage of PO_peak_, percentage of critical power, or percentage of maximal lactate steady state) might account for such differences (24). Whilst there is no complete explanation as to why women are less susceptible to fatigue than men when exercising within the severe-intensity domain, the reported lower level of peripheral fatigue in women might be attributed to some intrinsic sex differences in muscular traits and metabolic response to exercise. Since women have more type I muscle fibers (4), which are less fatigable than type II muscle fibers (25), peripheral fatigue might be naturally lower in them. Thus, our findings indicated that women experienced less neuromuscular fatigue and peripheral fatigue than men during a severe-intensity exercise performed until the limit of tolerance.

There was no reduction in VA after exercise in either men or women. Minor (5,26) or absent (15,27) reductions in VA have been reported after severe-intensity exercise, suggesting that neuromuscular fatigue in the severe-intensity domain is mainly determined by peripheral rather than central mechanisms (19). In addition, no sex difference in relation to central fatigue after severe-intensity exercise has been reported (5,26). Furthermore, a reduction in VA after an exercise task seems to occur in a similar manner for both men and women, regardless of exercise intensity, type of contraction (isometric or dynamic), or number of joints involved (2,28). Together, these findings suggested that mechanisms responsible for sex differences in neuromuscular fatigue in the severe-intensity domain are likely confined to contractile function traits.

Surface electromyography has some limitations as an indicator of muscle activation (29), but our findings indicated that while men progressively increased muscle activation from the beginning to the end of exercise, women had already presented high muscle activation at the beginning of the exercise and sustained this high muscle activation throughout the exercise. The higher muscle activation at the beginning of a cycling exercise in women than in men is in line with previous findings (6,7). In addition, the lack of increase in muscle activation from the beginning to the end of exercise in women and the progressive increase in muscle activation in men are also consistent with previously reported data comparing muscle activation pattern in women and men (6,7). Although the reason for this different pattern of muscle activation between women and men is not fully understood, it has been speculated that women might have a lower increase in neural activation of motor units already activated at the beginning of exercise, and consequently fatigued at the end of exercise, than men (30). On the other hand, as muscle activation is naturally high in the severe-intensity domain (31) and maximal muscle activation is nearly reached at failure of a severe-intensity exercise (26), women might have hastened their failure by starting with an already elevated muscle activation.

It is worth noting that the women in the present study presented lower M_wave_ than men. A lower M_wave_ in women than in men is in accordance with previous findings and might indicate lower membrane excitability in women (32). If women present lower membrane excitability, a higher muscle activation will be necessary to activate muscle fibers and sustain the external work rate. In addition, by presenting more type II muscle fibers (4), men might have been able to increase muscle activation progressively by recruiting type II muscle fibers during the last part of the trial and thus increase the time to failure. However, as type II muscle fibers are more fatigable, high intramuscular metabolic disturbance is expected (25,33), which may explain the greater peripheral fatigue in men than in women.

Finally, it is somewhat contradictory that women were more resistant to fatigue but had a shorter time to task failure than men. While it is hard to conciliate these findings, as discussed earlier and confirmed by previous findings (6,7), women presented higher muscle activation at the beginning of the cycling exercise. Increased muscle activation at the beginning of exercise indicates a higher muscle activation/power output ratio, which may explain the shorter time to failure in women. Furthermore, as we were unable to set exercise intensity based on individual critical power - the gold standard for determining the lower boundary of the severe-intensity domain (34) - the exercise intensity set by GET and PO_peak_ difference (i.e., Δ80) might have resulted in different relative exercise intensities within the severe-intensity domain between men and women. If women exercise at a higher relative intensity than men, a shorter time to failure is expected for women than for men. It could be argued that the relative difference in exercise intensity (within severe-intensity domain) between men and women in this study might affect the degree of neuromuscular fatigue and its central and peripheral components. Previous studies showed conflicting results when comparing the magnitude of neuromuscular fatigue and central and peripheral fatigue after different exercise intensities within the severe-intensity domain (20,35). While one study showed a similar degree of neuromuscular fatigue and its central and peripheral components during exercise close to the upper and lower boundaries of the severe-intensity domain (36), other studies showed higher peripheral fatigue with higher exercise intensity (20,35). Thus, future studies comparing women and men should standardize the exercise intensity using critical power.

### Strengths, limitations, and future directions

Our findings have practical implications for training prescription in women; the fact that women are less fatigable than men should be considered because lower skeletal muscle perturbation associated with peripheral fatigue may indicate lower training adaptations (5,37).

However, for the conclusions of the present study to be valid, women and men should have similar levels of sex-specific physical fitness. While the appropriate way to match physical capacity between women and men has not been established (38), the women and men who participated in the present study presented differences in relative V̇O_2max_ (∼14%) that are within the range reported for sex differences (10-16%) when training and talents are similar (10). In addition, relative V̇O_2max_ of men and women of the present study was similar when considering their sex (39), indicating that the two samples were well matched in terms of cardiorespiratory fitness. In addition, it could also be argued that women might have deliberately disengaged earlier than men in the time to task failure trial, which would explain the lower endurance performance, neuromuscular fatigue, and peripheral fatigue in women compared to men. This seems unlikely as women and men attained similar percentages of their V̇O_2max,_ HR_max_, and RPE_max_ at the end of the severe-intensity exercise, with values close to 100% for all these parameters.

Nevertheless, it is important to recognize some limitations of the present study. First, neuromuscular fatigue was assessed one minute after the end of the cycling exercise. Central and peripheral fatigue might continue to have a small but significant recovery within the first minute after exercise (32,35); therefore, neuromuscular fatigue could have been slightly underestimated. Nevertheless, the transition time from the cycle ergometer to the knee extension chair was the same in all experimental sessions, indicating that any potential underestimation in neuromuscular fatigue would have been similar in women and men. Second, the EMG activity of the antagonist muscles were not assessed during femoral nerve stimulation. Although previous control tests in our laboratory indicated minimal EMG activity of the knee flexors during femoral nerve stimulation, we cannot fully disregard activation of the knee flexors influencing supramaximal stimulation intensity. However, as we used 120% of the electrical stimulation intensity of the plateau in quadriceps twitch force and M_wave_ and the same protocol was applied in all experimental sessions, the influence of potential co-activation of antagonist muscles might have had minimal impact on our outcomes. Third, we were unable to run muscle biopsies; therefore, potential differences in the levels of intramuscular metabolic disturbance and the type of muscle fibers between men and women could not be determined. However, peripheral fatigue measured by potentiated twitch force is associated with intramuscular metabolic perturbation (33); thus, it is likely that women also experienced lower intramuscular metabolic perturbation than men. In addition, men presented a higher degree of neuromuscular fatigue, which may be an indication of more type II fibers in men than in women, as it has been demonstrated that individuals with greater proportion of type II muscle fibers exhibit greater resting twitch force but higher post-exercise decrease in twitch force than individuals with greater proportion of type I muscle fibers (40). This is also in line with studies showing a higher number of type II fibers in men than women (4).

Directions for further investigations also arise from the findings of the present study. Time to task failure in the present study was approximately 4-6 min, indicating that the work rate might have been in the upper boundary of the severe-intensity domain. Thus, further studies to test other exercise intensities (e.g., middle or bottom boundary) within the severe-intensity domain are desirable. Furthermore, studies determining the critical power to set the severe-intensity domain are also desirable. In addition, as neuromuscular fatigue and muscle activation might change during the menstrual cycle, further studies comparing different menstrual cycle phases would provide important insights into the physiology of women.

In conclusion, women had lower levels of neuromuscular and peripheral fatigue and greater muscle activation at the beginning of exercise during a severe-intensity cycling exercise than men.
